# The Importance of Humic Substances in Transporting “Chemicals of Emerging Concern” in Water and Sewage Environments

**DOI:** 10.3390/molecules28186483

**Published:** 2023-09-07

**Authors:** Anna Maria Anielak, Katarzyna Styszko, Justyna Kwaśny

**Affiliations:** 1 Faculty of Environmental Engineering and Energy, Cracow University of Technology, Warszawska 24, 31-155 Cracow, Poland; justyna.kwasny@pk.edu.pl; 2 Faculty of Energy and Fuels, AGH University of Science and Technology, Al. Mickiewicza 30, 30-059 Cracow, Poland; styszko@agh.edu.pl

**Keywords:** humic substances, micropollutants, pharmaceuticals, pesticides, sorption, activated carbon, ibuprofen, diclofenac, caffeine, carbamazepine, estrone, triclosan, bisphenol A, isoproturon

## Abstract

In this study, we examined the sorption of selected “chemicals of emerging concern” (CEC) on humic substances commonly found in water and municipal wastewater. These were ibuprofen, diclofenac, caffeine, carbamazepine, estrone, triclosan, bisphenol A, and isoproturon. The humic substances (HSs) were synthetic and not contaminated by the tested organic substances. The elemental composition and content of mineral micropollutants, gravimetric curves, and the IR spectrum of HSs were determined. We determined a relationship between the process efficiency and the characteristics of a sorbent and sorbate using the properties of organic substances sorbed on HSs. This relationship was confirmed by sorption tests on the HS complex, i.e., the HS-organic micropollutant. It has been shown that the given complexes have a greater affinity for hydrophobic surfaces than hydrophilic surfaces. To confirm the nature of the sorbent surfaces, we determined their zeta potential dependence on the pH of the solution. Studies have shown that HSs are carriers of both mineral substances and CEC in water and sewage environments.

## 1. Introduction

In 1993, an article was published on estrogen as an environmental pollutant [1]. Studies show that endocrine-disrupting chemicals are common in aquatic environments. Due to their multi-generational impact, even concentrations from μg/L to ng/L can negatively affect human health and the environment [2]. For over 20 years, impurities of various chemical classes, i.e., stimulants, sweeteners, active pharmaceuticals, cosmetic ingredients, and packaging materials, including classes of parabens, benzophenones and bisphenols, X-ray contrast media, pesticides, and industrial chemicals, could only be partially removed via conventional tertiary treatment technology [3,4]. In general, concentrations found in aquatic systems worldwide are generally low. However, ecotoxicological tests have shown that even low concentrations can cause subtle effects on biota [5]. All these substances have been classified as endocrine disruptors (EDCs) and can adversely affect many aquatic and terrestrial organisms. Typical EDCs are steroid estrogens, e.g., estradiol, estrone, estriol, xenoestrogens, alkylphenols, and bisphenol A. Other common and concerning examples of PPCP (pharmaceuticals and personal care products) are pharmaceuticals (ethynylestradiol, carbamazepine, and ibuprofen) and antimicrobials (triclosan) [6]. These are persistent organic pollutants, including compounds and toxic substances [7]. Research has shown [8] that ammonia monooxygenase is responsible for the biodegradation of triclosan. Triclosan binds to ammonia monooxygenase on the cell membrane and causes acute toxicity.

Triclosan up to 1.0 mg L^−1^ promotes metabolism unrelated to growth. However, a high concentration of about 4.0 mg L^−1^ is toxic to N. europaea cells. Many factors determine the persistence of micropollutants. Widely used active pharmaceuticals, cosmetic ingredients, and packaging materials, including classes of parabens, benzophenones, and bisphenols, can interact with organic carbon (OC) [9].

The authors Park et al. [10] tested 27 pharmaceuticals in liquid and solid-phase samples obtained from four different wastewater treatment plants. Studies have shown that nonsteroidal anti-inflammatory drugs (NSAIDs) predominate in wastewater. In four treatment plants, the bulk load of targeted pharmaceuticals was reduced by 88–95%, mainly through biological treatment. Among the biological processes, the removal efficiency was in the order of MBR > SBR > A_2_O > MBBR. The conventional wastewater treatment process reduces some organics and nutritional compounds from wastewater, and it results in toxicity reduction. Pharmaceutical residues and nanoparticles in STP effluents are some of the main micropollutants with estrogenic effect factors [11]. The authors of [12] found that the triclosan-resistant Pseudomonas aeruginosa G561870 strain could convert triclosan within 96 h of incubation. The biodegradation efficiency of triclosan was 99.89% ± 0.3 at a high concentration of 2 g/L by strain KS2002. Another study [13] showed that diclofenac was only partially removed through anaerobic biodegradation processes. The continuous release of diclofenac from wastewater treatment plants results in these compounds’ accumulation in the environment. At the same time, symptoms of hypersensitivity are caused by most nonsteroidal anti-inflammatory drugs. They cause shortness of breath in asthma patients and skin eruptions in urticaria patients. These symptoms are caused by indomethacin, mefenamic and flufenamic acids, ibuprofen, phenylbutazone, naproxen, diflunisal, zomepirac, and others [14].

In aquatic environments, the amount of pharmaceuticals can be minimized using a unique sustained-release drug delivery system based on osmosis, known as the elemental osmotic pump [15]. To remove diclofenac, the authors of [16] used sulfur-doped TiO_2_ (S-TiO_2_) compounds with reduced graphene oxide (rGO) and a weight percentage of rGO. However, they obtained different levels of effectiveness. Similar studies [17] have examined carbamazepine. One study investigated the photocatalytic degradation of the model pollutant carbamazepine under simulated solar irradiation with an N-doped TiO_2_-coated Al_2_O_3_ photocatalytic membrane using different water types. 

In [18], the authors investigated the use of living cells in the green algae Chlorella sp. to determine the short-term adsorption of ketoprofen (KET) and diclofenac (DIF) from aqueous solutions. The authors found that the bioremoval efficiency of KET and DIF was highly dependent on parameters such as the time, pH, algal dosage, and drug concentration. The adsorption efficiencies of both KET and DIC were maximized at pH 6. The maximum adsorption capacities of KET and DIF were 0.328 and 0.429 mg g^−1^, respectively.

The authors of [18] investigated ketoprofen (KET) and diclofenac (DIF) adsorption from aqueous solutions using living cells in the green algae Chlorella sp. The removal efficiency of KET and DIF depended on various indicators, such as the algae dose, process duration, pH, and drug amount. The maximum adsorption efficiency of both KET and DIC was at pH 6. The respective adsorption capacities of KET and DIF were reached at 0.328 and 0.429 mg g^−1^. These substances are partially biodegradable organic micropollutants. They migrate freely in the aquatic environment. They are mainly found in municipal sewage treatment plants, which also contain HSs. HSs are not even partially biodegradable; they adsorb on activated sludge, and lighter fractions, such as FA, are discharged with the treated sewage to surface waters. They are known to be carriers of both organic and mineral pollutants. HSs have a complex, aliphatic–aromatic structure. For this reason, they are carriers of organic substances. In the same way that they form complexes with metals, they can also form soluble complexes with organic substances.

Therefore, the aim of our study was to show that HSs are carriers of organic micropollutants and explain why HS micropollutant complexes are formed and how the HS structure affects the micropollutant sorption mechanism. Our final task was to test the effectiveness of removing HS micropollutant complexes from water with activated carbon.

## *2.* Results and Discussion 

### 2.1. Research on the Properties of HSs

We conducted a thorough qualitative analysis of the sorbent to study and explain the organic micropollutant sorption mechanism on HSs. The composition (C, N, H, O), inorganic micropollutants, and IR spectrum were determined. The elemental composition analysis in Table 1 indicates a high carbon and oxygen content at 55.34% and 38.13%, respectively, in the HS molecules. In contrast, the hydrogen and nitrogen content were lower at 5.19% and 1.34%, respectively. This elemental composition is typical of humic acids (HA and FA). The mass quotients of elementary elements calculated from the atomic weights are characteristic of soluble HA. The O/C quotient is small (0.52), which is an indicator of the oxidation degree of the molecule. The value is typical for aromatized FA, as well as for HA, which confirms that the H/C value (1.13) is slightly greater.

Table 2 shows the inorganic micropollutants in mg/kg of dry weight. The amount of inorganic impurities was significant, and the ash content was 38.13% m/m, including elements such as Na (1635 mg/kg), Ca (992 mg/kg), Cu (776 mg/kg), B (202 mg/kg), and Mn (165 mg/kg). Some amounts of Zn, Mg, Co, K, Fe, and Al were also found. The lowest were Ba (2 mg/kg) and Sr (6 mg/kg). In the processes of HS sorption, precipitation, and coagulation, cationic HS micropollutants are important because they participate as a result of bridging.

**Figure 1:**
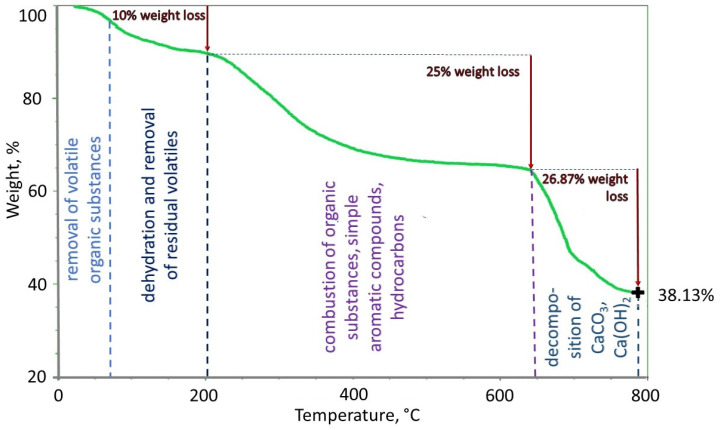
The TG curve of the HSs sample.

Figure 1 shows a thermogram (TG) of the temperature influence on the size of the sample mass. Our results show that in the range up to 200 °C, approximately 10% of the mass is removed. This mass includes transient and bound water and other volatile substances, including organic substances, that are already volatilized at low temperatures of 60–100 °C. In the temperature range of 200–650 °C, organic substances, simple aromatic compounds, carbohydrates, and hydrocarbons are burned and functional groups with a total mass of 25% of the initial sample mass are decomposed. Relatively large thermodegradation of organic substances occurs at temperatures >650 °C (up to 780 °C). In this temperature range, calcium carbonate (CaCO_3_) and Ca(OH)_2_ are decomposed, as indicated by a significant amount of Ca in the sample (992 mg/kg, Table 2). Other carbonates, such as iron carbonate, also decompose at high temperatures. The thermogram shows that the weight loss is 26.87% in the temperature range of 650–780 °C. At high temperatures, nitrogen compounds also decompose. In the case of the tested sample, its share of the initial sample (with ash content) was 0.83% m/m. The amount of ash, determined thermogravimetrically, is relatively high and amounts to 38.13%. Table 2 confirms the high ash content.

**Figure 2:**
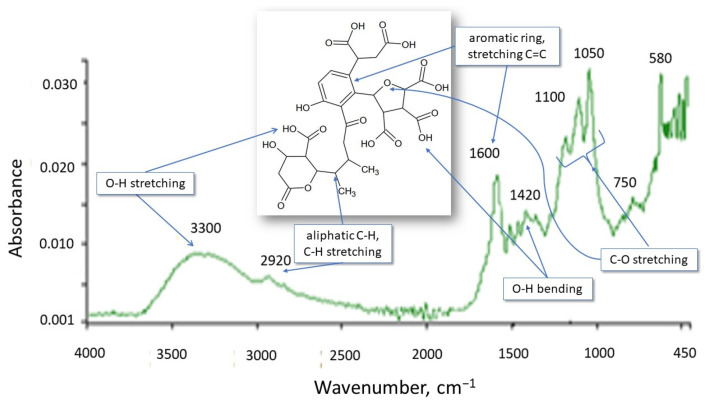
IR spectrum of HSs.

The HS structure, including functional group types, is important for assessing and analyzing the interactions between HSs and micropollutants. The appropriate inferences can be made in this area based on the IR spectrum. The spectrum of the tested HSs is shown in Figure 2. Comparing the spectrum to those found in the literature indicates that it is characteristic of FA and HA. Broad bands with peaks of 3300 and 2920 cm^−1^ result from the stretching vibrations of the O–H group and are characteristic of alcohols, phenols, and organic acids, confirming the aliphatic nature of HSs.

The presence of phenolic and carboxylic functional groups is confirmed by the simultaneous occurrence of the 1420 cm^−1^ peak resulting from O–H deformation, CH_3_ bending, OH phenolic stretching, and COO– antisymmetric stretching. The 1600 cm^−1^ band is characteristic of C=O, C=C double bonds (these are stretching vibrations) for ketones, aldehydes, esters, and alkenes in aromatic rings and indicates aromatic elements of the molecule. Peaks resulting from the stretching of C-O, C-O-C polysaccharides, alcohol groups, ethers, and phenols appeared in the area of 1200–1000 cm^−1^. The 750 cm^−1^ absorption band characterizes deformation vibrations in NO_2_ and NH_2_. In summary, these are HSs of an aliphatic–aromatic nature with negative functional groups (OH, COOH).

**Figure 3:**
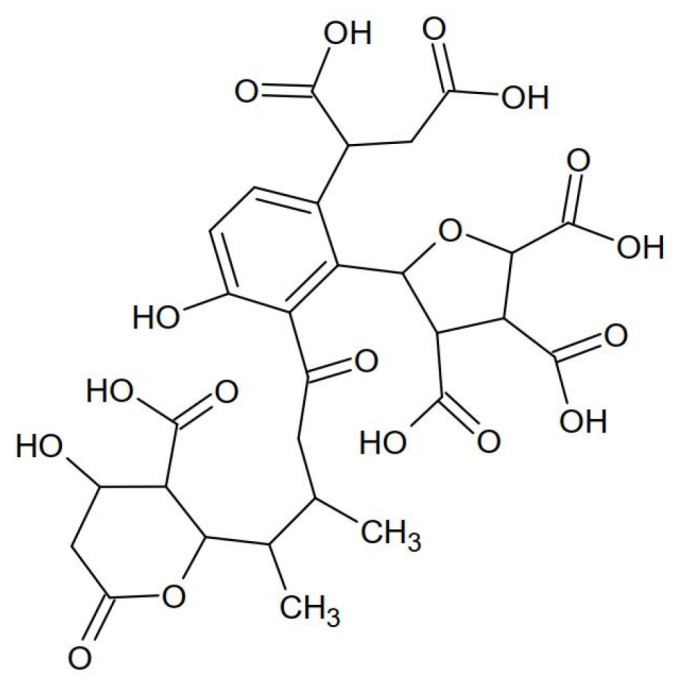
The structure of the HSs from the Suwannee River.

Studies of the IR spectra indicate high compliance between the structure of the tested HSs and the structure of the model developed by the authors [19] for the HSs extracted from the water of the Suwannee River (Figure 3). HSs have the same aliphatic functional groups and bonds as the internal hydrophobic structure.

### 2.2. Sorption Experiments with Pure HSs and Selected CEC

**Figure 4:**
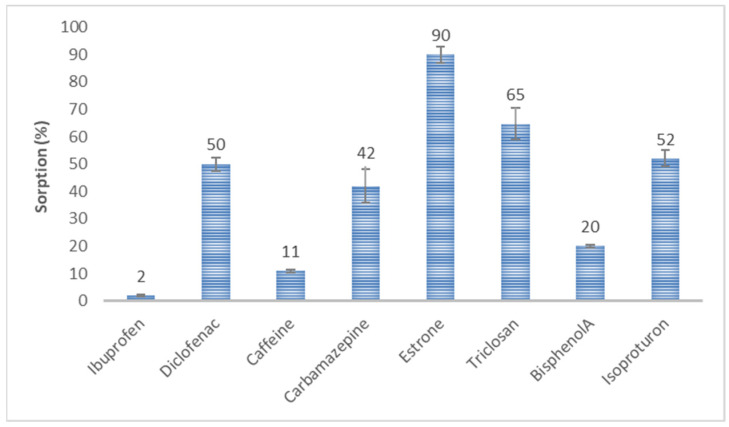
Sorption of selected organic substances on HSs.

Initial sorption experiments were conducted on pure HSs for eight CEC. Figure 4 shows the mean percentage of analytes sorbed after 4 h of shaking an HS solution of 10 mg/L with a single analyte at 1 μg/L, pH = 7.33. E1 had the highest sorption potential for HSs. On average, 90% of the compound was bound to the HSs. Then, an increase in the sorption potential for the tested HSs was observed for TCS, DCF, IPT, and CBZ at 40–65%. However, the sorption potential of CAF and BPA was 12 and 19%, respectively. IBF was persistent against bonding with the HSs.

Studies of the sorption process of CEC on HSs indicate a relationship between the sorption of organic micropollutants and their molar mass. The higher the molar mass of the CEC, the more efficient the sorption. This dependence can be presented in a series according to the amount of sorbed substance: 

(A) Ibuprofen (2%) < Caffeine (11%) < Bisphenol A (20%) < Carbamazepine (42%) < Diclofenac (50%) < Isoproturon (52%) < Triclosan (65%) < Estrone (90%)

or IBF < CAF < BPA < CBZ < DCF < IPT < TCS < E1

or in a series according to the size of the molar masses:

(B) Caffeine (194 g/mol) < Ibuprofen (206 g/mol) < Isoproturon (206 g/mol) < Bisphenol A (228 g/mol) < Carbamazepine (236 g/mol) < Triclosan (289 g/mol) < Estrone (270 g/mol) < Diclofenac (296 g/mol).

or CAF < IBF < IPT < BPA < CBZ< TCS < E1 < DCF

**Figure 5:**
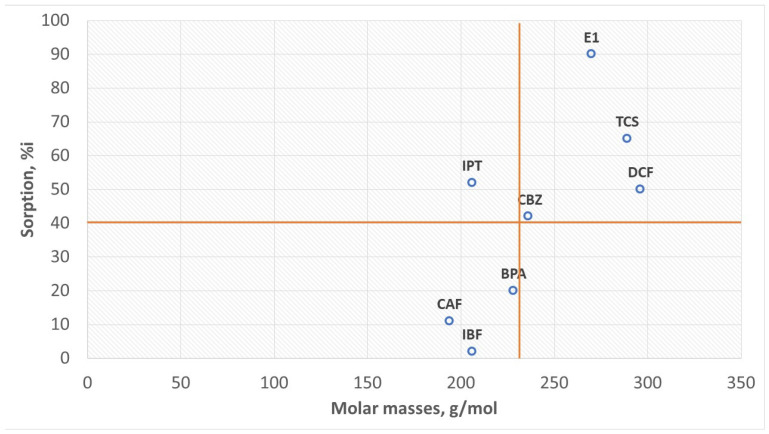
The influence of molar mass on sorption.

In series (B), the exception is IPT, which has a molar mass of 206 g/mol with 52% HS sorption, a pKa > 12 in water, has no acidic properties, and is a strongly conjugated hydroxide. The remaining compounds can be divided into two parts: a series of compounds with molar masses < 230 g/mol and HS sorption ≤ 20% and a second series of compounds with molar masses > 230 g/mol and HS sorption > 40% (Figure 5).

In most of the compounds analyzed, the greater the molar mass, the more developed the aromatic part. Therefore, the aromatic rings of these substances sorb on the aromatic HS surface. Targeted ionic sorption can also occur. For example, E1 sorption is the highest (90%), and its molar mass is lower than that of DCF whose sorption is 50%. E1 has hydroxyl (OH), ketone (C=O), and apolar methyl (CH_3_) groups. It can be a proton donor and an acceptor pole. E1 has extensive aromatic rings. Therefore, E1 sorption on HSs can be aromatic and ionic. DCF, with the highest molar mass, has carboxyl and amino groups. The amino group is a proton acceptor and separates the two aromatic rings, which may hinder apolar sorption. IPT has a lower molar mass than bisphenol A and nonpolar methyl groups, which increases its hydrophobic character. The organic complexes formed from van der Waals forces are durable enough to move in the aquatic environment and run off together with treated wastewater to surface waters. In this way, HSs resistant to biodegradation (mainly FA) become carriers of organic micropollutants and cause the migration of micropollutants to surface waters. On the other hand, HS aliphatic elements (Figure 3) are carriers of inorganic micropollutants in the qualitative analysis of the so-called ash. Consequently, if we want clean surface waters, we must remove HSs from municipal wastewater, which is the main source of the generation of organic and inorganic pollutants. The HS model presented in Figure 3 explains why these substances are good sorbents of electropositive mineral micropollutants as well as organic and hydrophobic substances. Electronegative substances show the weakest sorption. However, the process is very complex.

The smaller the log K_ow_, the more soluble the substance is in water. Such a substance is more polar. Consequently, this means that the higher the log K_ow_, the more soluble the substance is in fatty (nonpolar) substances. This substance is more hydrophobic.

**Figure 6:**
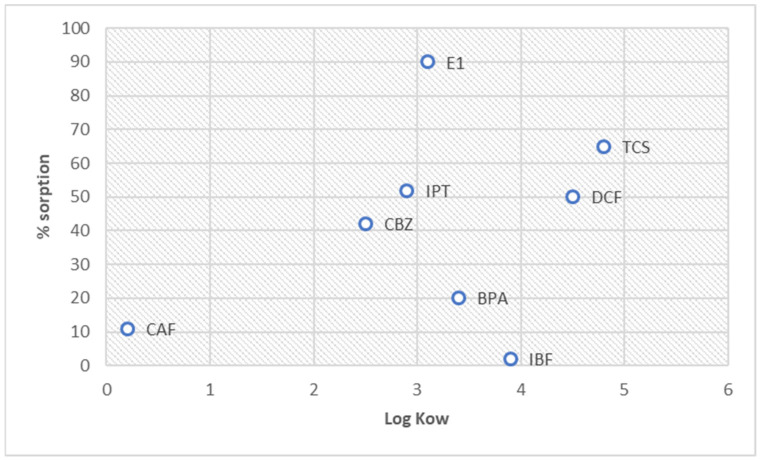
Graph of the K_ow_ value and the % sorption.

The high log K_ow_ values of compounds such as E1, DCF, TCS, IBF, and BPA suggest these analytes’ strong sorption hydrophobicity, which was found at the average level of sorption, except for IBF and BPA (Figure 6). DCF and IBF are anionic at the natural pH of the HS solution and affect the electrostatic interactions between the function groups of analytes and HSs. This finding implies that their sorption is less predictable. Furthermore, the difference in the sorption of BPA and E1 was analyzed with a similar log K_ow_. The structure of the compounds also impacts their interaction with HSs.

The relationship between the sorption and K_ow_ can be presented as a series:

For log K_ow_,

CAF < CBZ < IPT < E1 < BPA < IBF < DCF < TCS

For sorption,

IBF < CAF < BPA < CBZ < DCF < IPT < TCS < E1

The presented series does not indicate a clear correlation between HS sorption and the log K_ow_ index.

**Figure 7:**
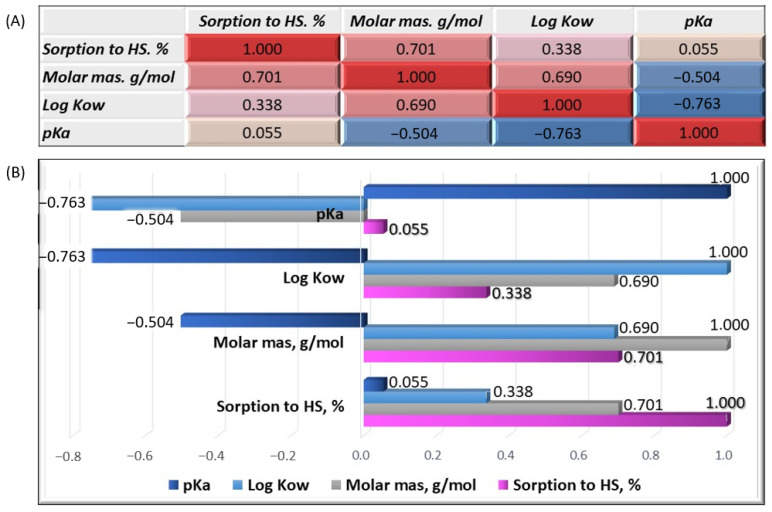
Correlation matrix of the analyzed variables (**A**) and graphical representation of the correlation coefficients (**B**).

We analyzed the correlation between the individual properties of organic micropollutants to verify our conclusions. A correlation matrix of variables, including the sorption efficiency on HSs, molar mass, logK_ow_, and pKa, was established. Figure 7A shows the resulting matrix. The positive correlations are marked in red and the negative correlations are marked in blue. The correlation coefficient is within the range <−1;1>. The correlation strength is greater the closer the coefficient value is to its extreme range. A value of −1 or 1 indicates perfect linear dependence. If the coefficient value is close to 0, it can be assumed that a correlation does not occur, and the analyzed variables are not correlated. The correlation strength was obtained using the color gradation of cells in the matrix. The coefficient of correlation for the HS sorption and molar mass was 0.701, allowing for a positive average correlation between these variables. Thus, our previous observations and conclusions about the importance of an organic substance’s molar mass for sorption efficiency were confirmed. The correlation coefficient of the HS sorption and logKow was 0.338, indicating a slight positive correlation between these variables. The correlation coefficient of the HS sorption and pKa was close to zero and amounted to 0.055.

Therefore, the sorption efficiency does not depend on the strength of the chemical compound (acid or conjugate base). Considering the correlation coefficients between the other variables, we found that the molar mass was positively correlated, on average, with the sorption efficiency and the octanol/water partition coefficient. Both the molar mass and logK_ow_ were negatively and moderately correlated with the pK_a_. Positive correlation coefficients were observed only for the HS sorption in all the variables (Figure 7B). 

The compounds that showed a sorption of >50% on HSs, such as E1, DCF, TCS, and IPT, were selected for further studies.

The absolute recoveries for E1, DCF, TCS, and IPT were 74% ± 13, 70% ± 5, 86% ± 7, and 63% ± 6, respectively, and the quantification limits were 42.1, 11.4, 5.6, and 2.7 ng/L, respectively.

### 2.3. IR Spectra and Interactions between HSs and Micropollutants

**Figure 8:**
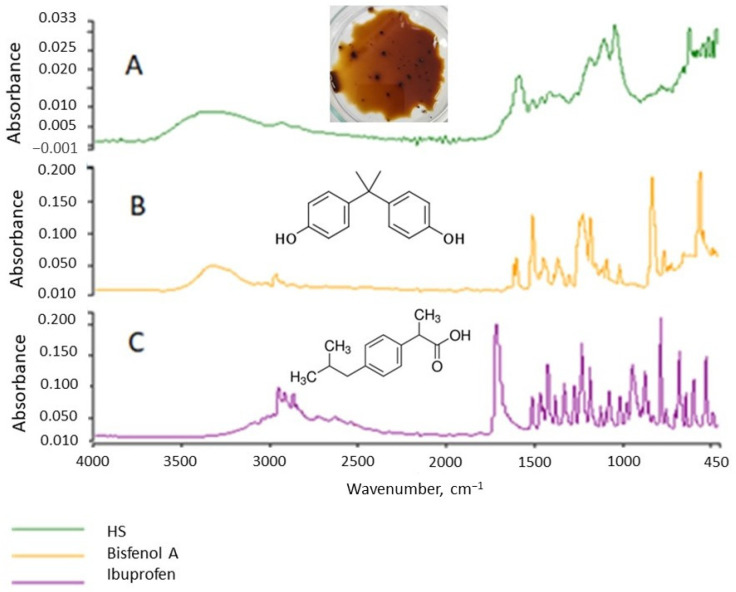
IR spectra of HSs (**A**), bisphenol A (**B**), and IBF (**C**).

**Figure 9:**
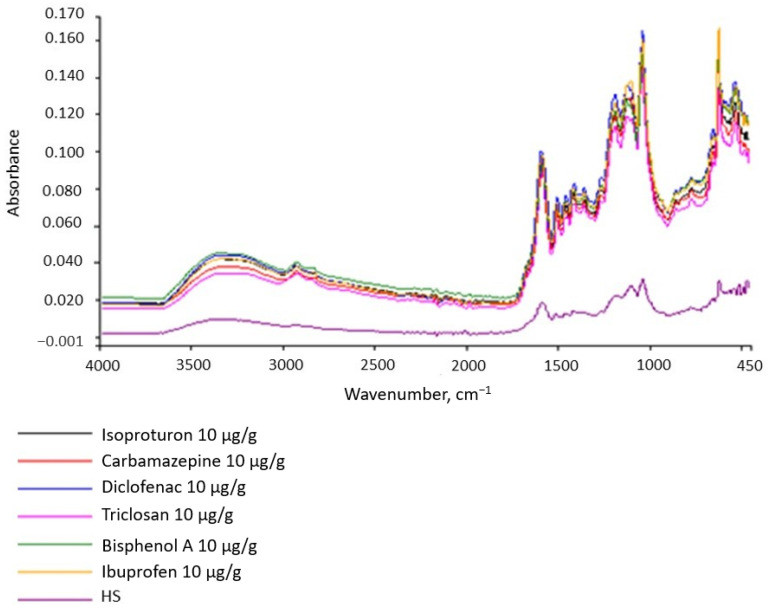
IR spectra of the tested substances.

HSs are almost always present in surface waters. The micropollutants present in water are sorbed on HSs and form organic complexes from them. To determine whether CEC affect the IR spectrum of HSs, the spectra of pure CEC were examined and compared to the HS spectrum (Figure 8). The spectra of the three organic substances, HSs, bisphenol A, and IBF were differentiated by their structures. Then, solutions of the CEC and HSs were prepared with several µg of CEC per 1 g of HS. These solutions were stirred for 4 h for the sorption of the CEC onto the HSs. After a given time, the IR spectra were determined (Figure 8). Figure 8 and Figure 9 present comparative analyses of the spectra, which show that the CEC affect the intensity of the HS bands but not their character. 

This observation is important because inorganic HS micropollutants are easy to analyze, whereas identifying organic micropollutants is difficult. Our research results indicate that they can affect the size of the absorbance, i.e., the intensity of the spectral bands. Therefore, caution should be exercised when inferring quantification from the absorbance value if the substance is not homogeneous. The amount of absorbance can inform the presence of micropollutants on the tested substance.

### 2.4. Sorption of CEC and HSs on Activated Carbon

Spectroscopy in ultraviolet and visible light UV/Vis is one of the methods used to study the structure of organic compounds and determine the number of micropollutants and degree of purity in solutions. We used the spectrum area of electromagnetic radiation from 200 nm to 780 nm for this spectroscopy research. The ultraviolet (UV) region ranges from 100 to 380 nm. For quantitative analysis, we used the near-ultraviolet region (wavelength 200–380 nm) and the visible part of the spectrum extending from 380 to 780 nm. The measured indicator was the transmittance of the electronic absorption spectrum (electron–oscillation–rotation) ranging from 0 to 100%.

**Figure 10:**
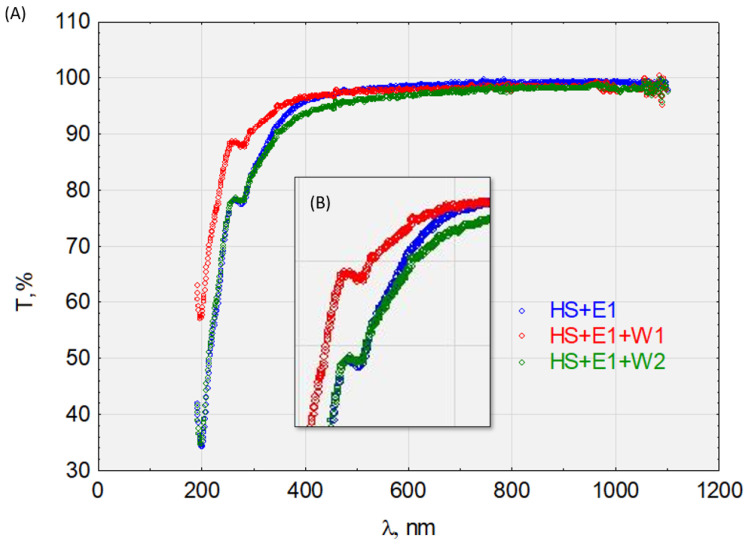
The transmittance of the solution with the complex HS + E1. (**A**) Before and after sorption on activated carbon W1 and W2 and (**B**) enlargement of the characteristic absorbance range.

**Figure 11:**
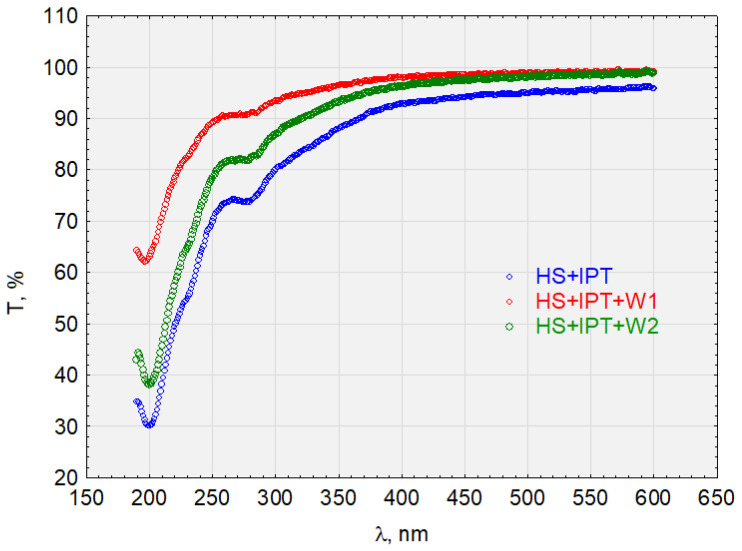
The transmittance of the solution with the complex HS + IPT before and after sorption on activated carbon.

**Figure 12:**
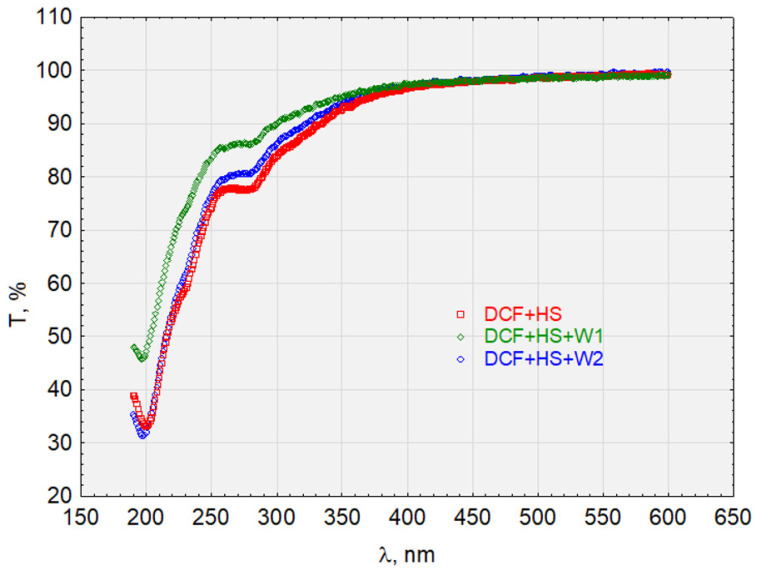
The transmittance of the solution with the complex HS + DCF before and after sorption on activated carbon.

**Figure 13:**
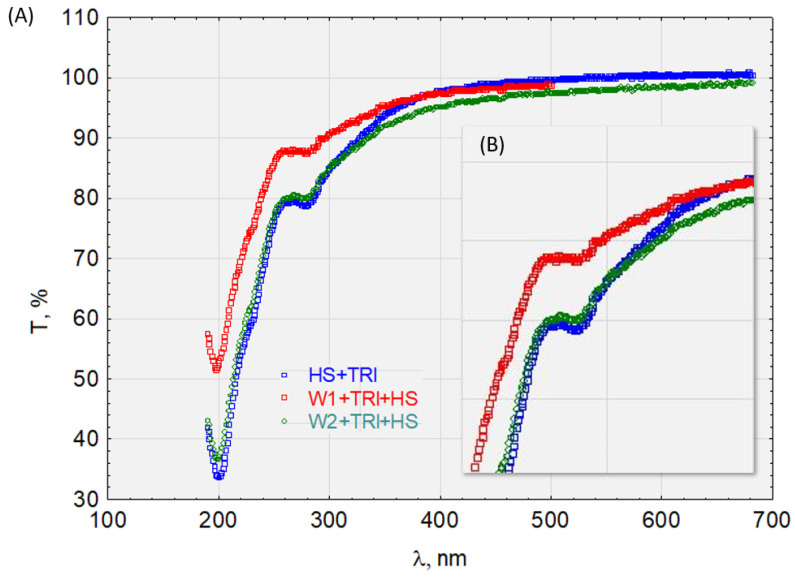
The transmittance of the solution with the complex HS + TRI (**A**) before and after sorption on activated carbon and (**B**) enlargement of the characteristic absorbance range.

To determine the sorption capacity of the HS complexes in the DCF, E1, TCS, and IPT, we determined the transmittance of the solutions before and after sorption on activated carbon W1 and W2 (Figure 10, Figure 11, Figure 12 and Figure 13). Figure 10 shows the graphical relationships for the HS + E1 complex solution before and after its sorption on W1 and W2. In the near-ultraviolet region, it can be assumed that there are no discrepancies between the course of HS + E1 and that obtained after the sorption process on hydrophilic carbon HS + E1 + W2. Small discrepancies occur in visible light, which may indicate the HS + E1 complex’s dispersive effect on hydrophilic carbon. However, the sorption results on hydrophobic carbon W1 are clearly favorable, and the transmittance for the examined spectrum at >190 nm is higher than for the solution HS + E1. Therefore, we can conclude that HS + E1 is sorbed on hydrophobic carbon and not hydrophilic carbon.

The HS + IPT complex (Figure 11) is sorbed on hydrophobic and hydrophilic activated carbon. However, there is a difference in the transmittance values in both the near-ultraviolet and visible light ranges, which are higher for sorption on hydrophobic carbon. Therefore, HS + IPT has a greater affinity for hydrophobic surfaces. For example, the transmittance at the wavelength λ = 250 nm for W1 was T = 90%, for W2 was T = 80%, and for the HS + IPT complex was T = 70%.

Similar relationships are seen in Figure 10 for the HS + E1 complex after analyzing the waveforms in Figure 12 for the HS + DCF complex. The HS + E1 complex has a greater affinity for hydrophobic carbon W1 than hydrophilic W2. Furthermore, the test results presented in Figure 13 indicate the activated carbon HS + TRI complex’s lack of sorption on W2 (hydrophilic) and its good sorption on hydrophobic carbon (W1).

**Figure 14:**
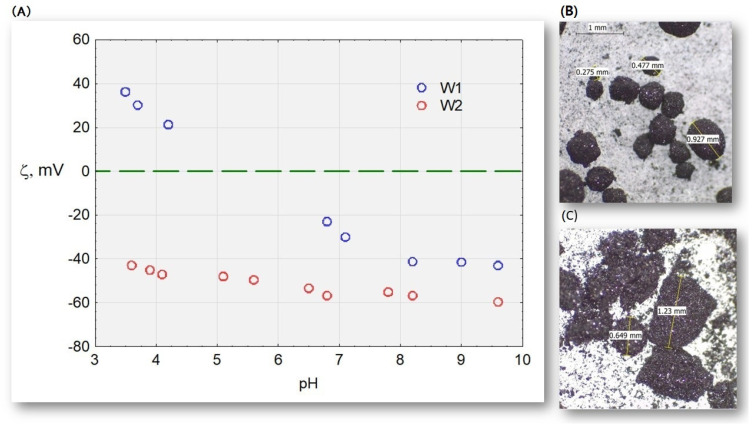
(**A**) Electrokinetic potential of activated carbons W1 and W2 and microscope photographs; (**B**) W1; and (**C**) W2.

Studies conducted in water on the zeta potential of two active carbons, W1 and W2, (Figure 14) at a pH range of 3.5–9.7 indicate that the hydrophilic activated carbon W2 in the tested pH range has negative values. Hydrophobic carbon is protonated with an excess of protons and has a positive potential in an acidic environment, whereas it has a negative potential (excess of OH- ions) in an alkaline environment. However, in an environment of cation–anion equilibrium, its potential is electroneutral. Research [20] has shown that the zeta potential of HSs extracted by the IHSS method ranges from −20 to −45 mV [20]. These values explain why there is no sorption of HS complexes on activated carbon. Electronegative activated carbon electrostatically repels negative HS complexes. However, in acidic and neutral environments, hydrophobic carbon has a surface easily accessible to HS complexes.

Further studies on DCF, E1, TCS, and IPT confirmed their high affinity for sorption on organic matter. The tested analytes’ concentration levels were below the level of quantification in all the samples. Double sorption processes after using HSs and activated carbon indicated that DCF and IPT were practically 100% sorbed by the tested materials. Analyte complexes are formed with HSs, and analyte residues that they are not bound to are adsorbed on activated carbons. Factors such as the chemical nature of the compounds, the ionization forms, the water solubility, and the properties of the sorbent determine their sorption on organic matter.

## 3. Materials and Methods

### 3.1. Materials

(a) HumiAgra humic substances (HSs) were used in this study. These are organic substances obtained by the hydrolytic and oxidative decomposition of lignosulfonates. HumiAgra substances are a mixture of FA and HA in the form of potassium salts. The solids were dissolved in water, and the 10 mg/L solution had a pH of 7.33. We used HumiAgra in our research because it did not contain organic micropollutants of anthropogenic origin, which were also used in our research and could affect the adsorption process by increasing the measurement error. Synthetic HSs that had no contact with organic micropollutants were used for the tests;

(b) We conducted analyses for selected CEC, which were diclofenac (DCF), ibuprofen (IBF), caffeine (CAF), carbamazepine (CBZ), estrone (E1), triclosan (TCS), bisphenol A (BPA), and isoproturon (IPT). Their formulas are presented in Table 1. Analytical standards with a purity of >98% were developed by Sigma Aldrich (St. Louis, MO, USA). The derivation agent BSTFA + 1% TMCS was obtained from Supelco (Bellefonte, PA, USA). Methanol, an analytical solvent, was obtained from Avantor Performance Materials Poland (Gliwice, Poland). The HLP5 system provided deionized water with a conductivity of <0.07 µS/cm (Hydrolab, Gdańsk, Poland). We used deionized water to prepare the samples for solid-phase extraction (SPE). HLB cartridges (a water-wettable polymer with a unique hydrophilic–lipophilic balance, 60 mg/3 mL) purchased from Waters (Wexford, Ireland) were used to extract organic substances from the liquid samples;

The organic substances used in our research were as follows: IBF (IUPAC name: (*RS*)-2-(4-(2-methylpropyl)phenyl)propanoic acid), DCF (IUPAC name: 2-[2-(2,6-dichloroanilino)phenyl)]acetic acid), CAF (IUPAC name: 1,3,7-trimethylpurine-2,6-dione), CBZ (IUPAC name: 5H-dibenzo[b,f]azepine-5-carboxamide 5H-dibenzo[b,f]azepine-5-carboxamide), E1 (IUPAC name: (3a*S*,3b*R*,9b*S*,11a*S*)-7-Hydroxy-11a-methyl-2,3,3a,3b,4,5,9b,10,11,11a-decahydro-1H-cyclopenta[a]phenanthren-1-one), TCS (IUPAC name 5-chloro-2-(2,4-dichlorophenoxy)phenol), BPA (IUPAC name: 4,4′-(propane-2,2-diyl)diphenol), and IPT (IUPAC name: 3-(4-Isopropylphenyl)-1,1-dimethylurea), whose chemical formulas are shown in Table 3.

IBF is a drug used to treat inflammation. It has analgesic and antipyretic effects and reduces swelling, blood clotting, and numbness in the joints, improving their mobility. DCF is a nonsteroidal drug with strong anti-inflammatory, analgesic, and antipyretic effects. CAF is a purine alkaloid found in coffee beans and several other plant materials. CBZ, a dibenzazepine derivative, is a psychotropic, anticonvulsant, and mood-stabilizing drug. E1 is a steroid, a weak estrogen (it is one of three major endogenous estrogens), and a minor female sex hormone. TCS is an antibacterial and antifungal agent present in some consumer products (e.g., toothpaste, soaps, detergents, and toys). BPA is a chemical compound primarily used in the manufacturing of various plastics. IPT is a herbicide used to control annual grasses and broad-leaved weeds in cereals.

(c) Activated carbon: hydrophobic activated carbon NORIT SA SUPER (W1), hydrophilic activated carbon NORIT CN (W2).

### 3.2. Methods

We determined the basic elements C, H, and N using the Flash 2000 elemental analyzer (Thermo Fisher Scientific, Waltham, MA, USA). The sample sizes were 5–10 mg. The ash content in the samples was tested using the thermogravimetric method. The SDT Q600 thermogravimeter by TA Instruments was used for the tests. The samples were heated to 700 °C (at a rate of 10 °C/min) with an airflow of 100 mL/min. The remaining elements were analyzed by X-ray fluorescence (XRF). 

We conducted the analysis using Brune’s ED-XRF spectrometer, Bruker’s S8 Tiger spectrometer, and Thermo Scientific’s EA Flash element analyzer using a powder analysis vessel on a 4 μm thick Proline film. The heavy metal content was determined using the Inductively Coupled Plasma Mass Spectroscopy (ICP-MS) method, in accordance with the PN-EN ISO 17294-2:2006 [21] standard.

We used Brune’s ED-XRF spectrometer, Bruker’s S8 Tiger spectrometer, and Thermo Scientific’s EA Flash element analyzer for the analysis along with a 4 μm Proline layer powder. The heavy metals were determined using inductively coupled plasma mass spectroscopy (ICP-MS) in accordance with PN-EN ISO 17294-2:2006.

Fourier-transform infrared (FTIR) spectroscopy with attenuated total reflection (ATR) was used to qualitatively identify the obtained FAs. We performed the tests in a range of 450–4000 cm^−1^ with a resolution of 1 cm^−1^ using a PerkinElmer spectrometer [22].

The samples containing IBF, DCF, CAF, CBZ, E1, TCS, BPA, and IPT were analyzed using a method described elsewhere [23]. Briefly, 100 mL of the sample was extracted with SPE cartridges and sequentially conditioned with 2 mL of methanol and 2 mL of deionized water. The analytes were eluted with 4 mL of methanol. The resulting extract was evaporated to dryness in a stream of nitrogen. The dry residue was redissolved in 200 µL of a derivatization reagent and the silylation reaction was conducted at 60 °C for 30 min. The supernatants were transferred into 1.5 mL autosampler vials for analysis by GC-(IT)MS/MS (Thermo Scientific Trace 1310 gas chromatograph with a Tri Plus RSH autosampler coupled with an ion trap mass spectrometer ITQ 900). Blank and control samples (without fulvic acid) containing 1 µg/L of the micropollutant were analyzed after being prepared using the same procedure as described above. The experiments were performed in triplicate. The respective solutions were analyzed by duplicate injections and all the values for the respective sorption experiment were averaged.

We used a ZetaPALS (Brookhaven Instruments Co., Holtsville, NY, USA) instrument to determine the zeta potential with a head-on analysis of scattered laser light. Deionized water with a maximum specific conductivity of 1.5 μS/cm was used to prepare the solution.

The sorption of the CEC was conducted by shaking the HS solution with the CEC for 10 min at a speed of 150 rpm at a temperature of 25 °C. The concentration of adsorbed CEC was 1 µg/L. The compounds (E1, DCF, TCS, and IPT) that showed a high sorption potential for HS acids at >50% were selected for further studies. These studies consisted of shaking E1, DCF, TCS, and IPT solutions at a concentration of 1 µg/L with an HS concentration of 10 mg/L for 4 h at 25 °C and then for 120 min with activated carbon.

## 4. Conclusions

The sorption mechanism of micropollutants on HSs is explained in this manuscript. Studies show that CEC with a high molar mass of >230 g/mol have a high affinity for the surface of HSs. Functional groups can also influence the sorption of HSs. E1 (270 g/mol, 90%), TCS (289, g/mol, 65%), DCF (296 g/mol, 50%), and CBZ (235 g/mol 42%) were best sorbed on the HSs. CAF (194 g/mol, 11%) and IBF (206 g/mol, 2%) showed the lowest sorption.

The complexes HS + E1, HS + DCF, and HS + TRI showed a high affinity for hydrophobic carbon (W1) and were not sorbed on hydrophilic activated carbon (W2). The HS + IPT complex was sorbed on W2 but was better sorbed on W1. The sorption efficiency is determined by the ionic nature of the complexes and especially the surface charge of the HSs and activated carbon. These relationships are confirmed by zeta potential measurements.

CEC sorbed on HSs are transported differently based on their chemical properties and structures. The transportation of CEC predominantly occurred for E1 and TRI, where there was a hydrophobic character. Compared to sorption by other processes, hydrophilic substances are not relevant. Based on the conducted tests, we can conclude that inorganic substances are sorbed by aliphatic groups and organic substances are mainly sorbed by HS organic parts.

Our research results indicate that they can affect the size of the absorbance, i.e., the intensity of the spectral bands. Therefore, caution should be exercised when inferring quantification from the absorbance value if the substance is not homogeneous. We recommend using sorption on hydrophobic carbon to remove HSs and complex HS micropollutants from water. 

## Figures and Tables

**Table 1:** The elemental composition of HSs applied in the research.

Elementals Content Ash Free (%)					Atomic Ratios (-)			
C	H	N	O	Ash	O/C	H/C	C/N	O/H
55.34	5.19	1.34	38.13	38.13	0.52	1.13	4.80	0.46

**Table 2:** Mineral components in HSs.

Element	Result	Unit
Al	31	mg/kg
B	202	mg/kg
Ba	2	mg/kg
Co	101	mg/kg
Ca	992	mg/kg
Cu	776	mg/kg
Fe	47	mg/kg
K	76.41	mg/kg
Mg	150	mg/kg
Na	1635	mg/kg
Mn	165	mg/kg
Sr	6	mg/kg
Zn	157	mg/kg
Ash	38.13	%(m/m)

**Table 3:** CEC applied in the research.

Ibuprofen Formula: C_13_H_18_O_2_ Molar mass: 206 g/mol Kow = 3.9 pKa = 4.4	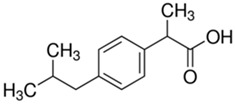	Diclofenac Formula: C_14_H_11_Cl_2_NO_2_ Molar mass: 296 g/mol Kow = 4.5 pKa = 4.2	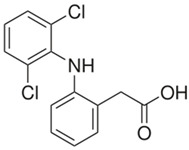
Caffeine Formula: C_8_H_10_N_4_O_2_ Molar mass: 194 g/mol Kow = 0.2 pKa = 14	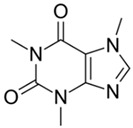	Carbamazepine Formula: C_15_H_12_N_2_O Molar mass: 236 g/mol Kow = 2.5 pKa = 13.9	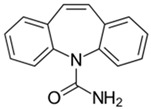
Estrone Formula: C_18_H_22_O_2_ Molar mass: 270 g/mol Kow = 3.1 pKa = 10.3	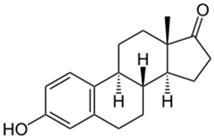	Triclosan Formula: C_12_H_7_Cl_3_O_2_ Molar mass: 289.54 g/mol Kow = 4.8 pKa = 7.7	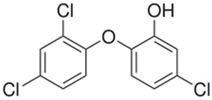
BisfenolA Formula: C_15_H_16_O_2_ Molar mass: 228.29 g/mol Kow = 3.40 pKa = 10.3	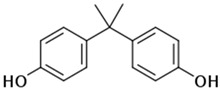	Isoproturon Formula: C_12_H_18_N_2_O Molar mass: 206 g/mol Kow = 2.5 pKa = 13.8	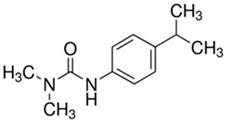

## Data Availability

The data presented in this study are available on request from the corresponding author.
